# Comprehensive Assessment of Prognostic Factors for Immune-Related Adverse Events in Immune Checkpoint Inhibitor-Treated Melanoma

**DOI:** 10.3390/cancers17172806

**Published:** 2025-08-27

**Authors:** Julian Kött, Myriam Merkle, Lina Bergmann, Noah Zimmermann, Tim Zell, Isabel Heidrich, Glenn Geidel, Klaus Pantel, Stefan W. Schneider, Christoffer Gebhardt, Daniel J. Smit

**Affiliations:** 1 Department of Dermatology and Venereology, University Medical Center Hamburg-Eppendorf, 20246 Hamburg, Germany; j.koett@uke.de (J.K.); t.zell@uke.de (T.Z.); i.heidrich@uke.de (I.H.); g.geidel@uke.de (G.G.); st.schneider@uke.de (S.W.S.); 2 Fleur Hiege Center for Skin Cancer Research, University Medical Center Hamburg-Eppendorf, 20246 Hamburg, Germany; pantel@uke.de; 3 Institute of Tumor Biology, University Medical Center Hamburg-Eppendorf, 20246 Hamburg, Germany; li.bergmann@uke.de

**Keywords:** melanoma, immune-related adverse events, irAE, immune checkpoint inhibition, D-dimers, prognosis

## Abstract

**Background**: Immune checkpoint inhibition (ICI) is the standard treatment for advanced melanoma patients. Despite its high efficacy compared to previous treatment options, immune-related adverse events (irAEs) occur frequently. While most of the patients experience mild to moderate irAEs, some patients develop severe to lethal irAEs under ICI treatment; hence, biomarkers are urgently required. **Methods**: In this retrospective single-center study, 157 advanced melanoma patients treated with ICI at the University Medical Center Hamburg–Eppendorf were included. IrAEs were correlated with clinico-pathological parameters, disease-related outcomes, and irAE-free survival. **Results**: In our cohort, 130 out of 157 patients receiving immunotherapy experienced irAE, of which more than half experienced irAE Grade ≥ 3. The most common irAE independent of its grade included cutaneous irAE, colitis, endocrine irAE, and hepatitis. Patients experiencing irAE had significantly longer progression-free survival (PFS) and overall survival (OS) compared to patients who did not experience irAE under ICI therapy. Stratification by irAE groups revealed that musculoskeletal irAEs are associated with the longest, whereas myocarditis is associated with the shortest OS and PFS. IrAE was a significant beneficial prognosticator for PFS in univariate, but not in multivariate Cox regression analysis. With respect to OS, the occurrence of irAE was an independent prognostic factor among ECOG status ≥ 2 and uveal melanoma. ROC analysis demonstrated that D-dimers have moderate predictive capability for irAE occurrence. Cox regression analysis demonstrated that elevated D-dimers and PD-1 monotherapy vs. CTLA-4 and PD-1 combination regimen are the only independent prospective prognostic markers for irAE-free survival. **Conclusions**: Our study demonstrates that different irAE across the irAE spectrum have a different impact on the PFS and OS of advanced melanoma patients. D-dimers may be used as a blood-based biomarker for irAE prediction, warranting future validation in multi-center studies.

## Simple Summary

Patients with melanoma, a malignant cancer of the skin, are treated with so-called immune checkpoint inhibitors that can reactivate the immune system to destroy the tumor cells. However, immune-related adverse events (irAEs) during the treatment with immune checkpoint inhibitors are frequent and sometimes even life-threatening. In this work, we comprehensively assessed the impact of the diverse range of side effects that can occur with respect to the outcome of melanoma patients. It is already known that irAEs indicate treatment response, but it is still unclear if this is true for all irAEs. We observed that specific, but not all, treatment-associated side effects are associated with a favorable prognosis and survival in melanoma. Moreover, we have identified D-dimers, which are small protein fragments that are released when blood clots break down in the body, as a potential blood-based biomarker that can predict who will experience irAEs.

## 1. Introduction

The incidence of malignant melanoma has increased continuously in recent decades and is currently one of the most common tumors in Caucasians. Moreover, in contrast to other solid tumors, melanoma occurs frequently in young and middle-aged adults [1]. A few years ago, the presence of distant metastases was associated with a median overall survival (OS) of less than one year [2]. With the introduction of immune checkpoint inhibitors (ICI), there has been significant progress in the treatment of metastatic melanoma [3]. The current standard of care for patients with metastatic melanoma is a combined therapy with cytotoxic T-lymphocyte-associated protein-4 (CTLA-4) antibody ipilimumab and programmed cell death protein (PD-1) antibody nivolumab, with 5-year OS rates of 52% [3]. Unfortunately, not all patients benefit from these treatments, and a significant number of patients with metastatic melanoma will progress on ICI due to treatment resistance [4].

Another limiting factor in the treatment with ICIs is the occurrence of immune-related adverse events (irAEs). ICIs block inhibitory checkpoints and thereby activate the T-cell-mediated immune response by reducing the co-inhibitory signals. This results in a non-specific activation of the immune system, which disrupts immunological homeostasis and reduces T-cell tolerance [5]. IrAEs can affect any organ system and strongly vary in severity, ranging from irAEs that do not require treatment to irAE-associated mortality in melanoma patients [5,6]. The Common Terminology Criteria for Adverse Events (CTCAE) represent a catalog for ranking of irAE in severity grades 1 to 5, whereas grade 1 represents asymptomatic or mildly symptomatic irAEs and grade 5 represents AE-related mortality. Among the irAEs, gastrointestinal, endocrine, and dermatological irAEs are most frequently observed. Neurotoxicity and cardiotoxicity occur less frequently but are more often severe. In addition, irAEs are more common during treatment with the combination of a PD-1 with a CTLA-4 antibody compared to monotherapy [6,7]. In the Checkmate-067 study, which led to the approval of the combined therapy with ipilimumab and nivolumab, almost all patients (96%) treated with ipilimumab plus nivolumab experienced irAEs, of which 59% were grade 3–4, and 42% led to an interruption of the treatment. Moreover, two deaths were reported that were associated with treatment-related AEs [7,8].

In general, the management of irAEs differs from chemotherapeutic side effects. In the case of mild irAEs (grade 1), treatment can usually be continued under close monitoring. In the case of moderate irAEs (grade 2), treatment with ICI should initially be paused and treatment with corticosteroids started. In severe irAEs (grade 3 and above), high-dose steroid therapy should be performed over 4–6 weeks. If there is no improvement after 48–72 h of high-dose steroid treatment, the use of TNF-α inhibitors such as infliximab should be considered. As soon as symptoms and laboratory values indicate recovery and the irAE is reclassified as grade 1, treatment with ICI can be resumed [9]. If life-threatening side effects occur (grade 4), this usually leads to a permanent discontinuation of the treatment. A central question is whether there is a connection between the occurrence of irAEs and the effectiveness of ICI, as their presence proves an activation of the immune system. However, whether these lead to better response or are even suitable as prognostic markers for therapy response remains controversial [5]. Interestingly, the occurrence of vitiligo as an irAE appears to be associated with an improved prognosis for OS and PFS in melanoma treated with PD-1 antibodies [10,11].

IrAEs are one of the central factors limiting treatment with ICIs. Their burden is particularly high as they occur frequently, are often severe, and have a great impact on the quality of life and prognosis [12]. There are currently no approved biomarkers for clinical practice that predict the occurrence and severity of irAEs [13,14]. Furthermore, established biomarkers that can be used in decision-making to determine which patients benefit from monotherapy with a PD-1 antibody and which benefit from combined therapy are lacking. This is particularly relevant regarding the higher level of toxicity of combined therapy [15]. Ideally, this biomarker could be used additionally in decision-making for *BRAF*-mutated melanoma between ICI and BRAF or MEK inhibitor treatment, and contribute to the further optimization of personalized medicine in dermatological oncology.

## 2. Materials and Methods

### 2.1. Study Population

In this single-center, retrospective observational study, a total of 157 melanoma patients treated for advanced melanoma between 2017 and 2023 at the Department of Dermatology and Venerology, Skin Cancer Center at the University Medical Center Hamburg–Eppendorf were included. All patients received immunotherapy with ICIs, either as anti-PD-1 monotherapy with nivolumab or pembrolizumab, or as an anti-PD-1/anti-CTLA-4 combination therapy with nivolumab and ipilimumab. The American Joint Committee on Cancer (AJCC) stage (8th edition) and Eastern Cooperative Oncology Group (ECOG) performance stage were documented in addition to demographic, clinical, and histopathological data, as well as a detailed description of the type and grade of irAEs according to CTCAE. Lactate dehydrogenase (LDH), S100B, D-dimers, and C-reactive protein (CRP) levels, as well as leukocyte, neutrophil, and lymphocyte counts, were extracted from clinical records. The following thresholds were applied to define the parameters as elevated: LDH—246 U/L; S100B—0.15 µg/mL; D-dimers—0.52 mg/L; CRP—5 mg/L. S100B, D-dimer, and CRP values below the lower limit of detection (LoD) were included as metric values with half of the lower limit of detection (LoD S100B: 0.021 µg/mL; LoD D-dimer: 0.19 mg/L; LoD CRP: 5 mg/L).

For further analysis, the documented irAEs were grouped as follows: Endocrine irAE: hypophysitis, thyreoiditis, pancreatitis, diabetes mellitus; cutaneous irAE: dermatitis, vitiligo, lichen ruber, alopecia; musculoskeletal irAE: arthritis, myositis; neurological irAE: neuritis, myelitis, myasthenia; other irAE: anemia, sarcoid-like reaction, fatigue, nephritis, lymphopenia, siladenitis, gastritis, panniculitis, infusion reaction, uveitis, mucositis, vasculitis, Sjörgen-like syndrome. Therapy progression was defined according to Response Evaluation Criteria in Solid Tumors (RECIST) version 1.1 [16] when based on radiological assessment. The response categories are classified as complete response, partial response, stable disease, and progressive disease (PD). In case of local relapse, clinical/pathological assessment was used to define PD. Patients with disease progression within six months after therapy start were defined as primary resistant, and patients with progression more than six months after therapy start as secondary resistant. SD was counted as a response. Only PD was counted as a PFS event for outcome analysis. All patients provided written informed consent to study participation, and the study protocol was approved by the ethics committee of the Hamburg Medical Association (PV5392).

### 2.2. Statistical Analysis

RStudio (Posit PBC, Boston, MA, USA) version 2024.09.0+375 with R version 4.4.1 and the packages finalfit (v.1.0.8), survival (v.3.7-0), and survminer (v.0.4.9) were used for statistical analysis. Intergroup comparison of categorical variables was performed with Fisher’s exact test. Continuous variables were tested for normality using the Shapiro–Wilk test. Additionally, for parametric data, homoscedasticity was assessed by Levene’s test. For intragroup comparisons of parametric variables with equal variance, a two-sided T-test (2 groups) or ANOVA (>2 groups) was used. In case of heteroscedasticity, the Welch T-test (2 groups) or the Welch ANOVA (>2 groups) was applied. For intergroup comparison of non-parametric data, the Kruskal–Wallis test was used. PFS, OS, and irAE-free survival after therapy start are depicted as Kaplan–Meier plots and compared by log-rank test (Mantel–Cox). In Kaplan–Meier plots, including more than two groups, pairwise comparisons were conducted using the log-rank test followed by adjustment for multiple testing using the Benjamini–Hochberg correction method. Univariate Cox regression analysis was used to compare the prognostic value of clinico-pathological parameters with respect to PFS, OS, and time to occurrence of irAE. Only variables with significant effects in univariate analysis were included in the multivariate Cox regression analysis. Receiver operating characteristic (ROC) curves were calculated and plotted with pROC (v.1.18.5). Statistical significance was defined as a *p*-value < 0.05.

## 3. Results

### 3.1. Clinical Cohort

To assess the occurrence of irAE under ICI and identify prognostic factors, we collected comprehensive clinico-pathological data from 157 advanced melanoma patients treated at the University Medical Center Hamburg–Eppendorf. At the start of therapy, patients had a mean age of 66 years, and the majority showed a good performance status of ECOG 0. Most patients had the diagnosis of stage IV melanoma, with superficial spreading melanoma and nodular melanoma being the most common histological subtypes. Distant metastatic sites included the lung (78 out of 121 patients, 64.5%), distant lymph nodes (50 out of 121 patients, 41.3%), the liver (38 out of 121 patients, 31.4%), and the brain (32 out of 121 patients, 26.4%). Patients were either treated with anti-PD-1 monotherapy (nivolumab or pembrolizumab) (29.3%) or anti-CTLA-4 + anti-PD-1 (ipilimumab + nivolumab) combination therapy (70.7%). Most patients were treated with first-line therapy (73.9%). Among the 41 patients that received previous therapies before inclusion, most frequently anti-PD-1 monotherapy (pembrolizumab or nivolumab) (61.0%), followed by BRAF/MEK inhibitors (26.8%), and interferon therapy (12.2%) was administered (Table 1).

### 3.2. IrAE Spectrum

We observed irAEs in 82.8% (*n* = 130) of all patients treated with ICI. In total, 71.5% (*n* = 93) of patients with irAEs first experienced mild to moderate irAEs (CTCAE grade 1–2) that did not require hospitalization, while 52.3% (*n* = 68) suffered from severe (CTCAE grade 3) to life-threatening (CTCAE grade 4) irAEs during the course of their treatment. The number of irAEs per patient in the group of patients that experienced irAE ranged from 1 to 6, with a median number of 1.5 (IQR: 1–3 irAEs) occurring per patient (Table 2). The median time until the occurrence of the first irAE was 41 days (IQR: 22–70 days) after treatment initiation.

A grouped description of the type and grade of irAE is provided in Table 3. The most commonly observed irAE within the patient group that experienced irAE (*n* = 130) was cutaneous irAEs (46.9%, *n* = 61) that mostly occurred at mild or moderate severity (93.4%, *n* = 57) and did not require hospitalization. Colitis (41.5%, *n* = 54), endocrine (33.8%, *n* = 44), hepatitis (32.3%, *n* = 42), other irAEs (31.5%, *n* = 41), and musculoskeletal irAEs were less common (22.3%, *n* = 29) within the group that experienced irAEs, but still occurred frequently. For endocrine and musculoskeletal irAEs, the majority of patients experienced mild to moderate irAEs (77.5%, *n* = 35, and 85.7%, *n* = 25); however, for hepatitis, more than half of the irAE-affected patients experienced severe to life-threatening irAEs (54.8%, *n* = 23). Patients experiencing irAEs rarely had neurological irAEs or irAE-induced myocarditis (both *n* = 4 (3.1%)). While neurological irAEs mostly had a low to moderate grade, immunotherapy-related myocarditis was often graded severe to life-threatening (75.0%, *n* = 3). A more detailed description of the type of irAE and its associated grades is presented in Supplementary Table S1. Moreover, we have assessed whether the occurrence of subtypes of irAE is dependent on sex, but only found a significant association for hepatitis (*p* = 0.023) with a more frequent occurrence in female patients (Supplementary Table S2). Stratification for major subtypes (i.e., CUP, mucosal melanoma, ocular melanoma, and cutaneous melanoma) included in this study revealed a significant association of the subtype with the occurrence of pneumonitis (*p* = 0.034) (Supplementary Table S3).

### 3.3. Association of Clinico-Pathological and Laboratory Parameters with irAEs

Next, we analyzed several established clinico-pathological parameters with respect to the occurrence of irAEs (Table 4). No significant differences were observed with regard to the sex (*p* = 0.512) and age (*p* = 0.059) of the patients, as well as the melanoma subtype (*p* = 0.945), while low ECOG status was significantly associated with the occurrence of irAEs (*p* = 0.002). AJCC stage (*p* = 1.000) showed no difference in patients with and without irAEs. While the therapy line was not associated with irAE occurrence (*p* = 0.925), patients treated with combination therapy more frequently suffered from irAEs (*p* < 0.001). None of the tissue mutations analyzed during routine diagnostics were associated with the occurrence of irAEs. When we further divided the patients into subgroups (grade 1–2 and grade ≥ 3), we observed similar differences in ECOG status (*p* = 0.010) and therapy type, with more severe irAEs in patients treated with combination therapy (*p* < 0.001) (Supplementary Table S4).

In addition, we assessed several routine diagnostic laboratory markers, including LDH, S100B, D-dimers, CRP, lymphocytes, leukocytes, neutrophils, and the neutrophil-to-lymphocyte ratio (NLR), before therapy started to identify potential predictive markers for the occurrence of irAEs (Table 5). For LDH (*p* = 0.025), S100B (*p* = 0.031), and D-dimers (*p* = 0.002), reduced levels were observed at baseline in patients who developed irAEs compared to the patients who did not develop irAEs. Further comparison of these markers between patients with no, mild to moderate (grade 1–2) and severe to life-threatening (grade ≥ 3) irAEs showed a similar reduction for D-dimers in both grade groups compared to patients without irAEs (*p* = 0.009), whereas no significant differences were observed for LDH (*p* = 0.069) and S100B (*p* = 0.092) in this subgroup analysis (Supplementary Table S5).

### 3.4. PFS and OS Depending on the Occurrence and Subtype of irAEs

**Figure 1:**
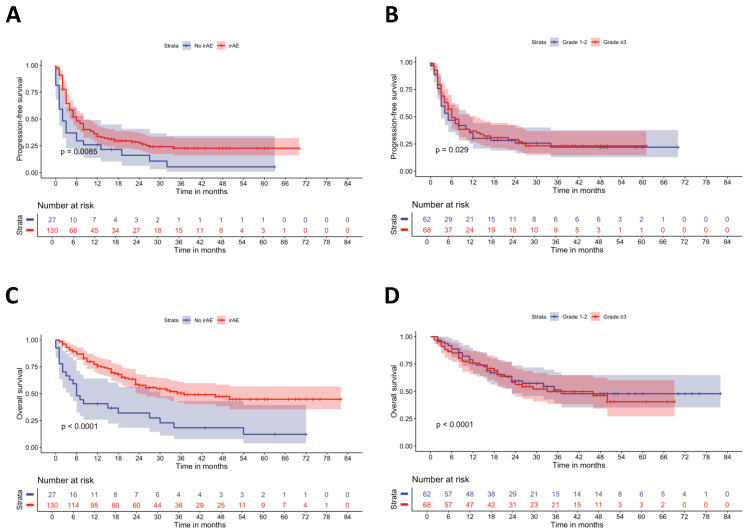
Kaplan–Meier survival plots of progression-free and overall survival of melanoma patients depending on the occurrence of irAE and the grade of the strongest irAE. (**A**) Progression-free survival stratified by the occurrence of any irAEs. (**B**) Progression-free survival stratified by the grade of the strongest irAE. (**C**) Overall survival stratified by the occurrence of any irAEs. (**D**) Overall survival stratified by the grade of the strongest irAE. Statistical analysis for (**A**–**D**) was performed using the log-rank test.

In the next step, we assessed PFS and OS outcomes in our patient cohort depending on the occurrence of any irAEs and in a subtype-specific manner. Regarding PFS, patients who experienced any kind of irAE had a significantly higher PFS, with a median time to progression of 6 months (95% CI: 5–8) compared to 2 months (95% CI: 1–8) in patients without irAEs (*p* = 0.0085) (Figure 1A). Further stratification of patients with low to moderate and severe to life-threatening irAEs confirmed the differences depending on the irAE grade regarding PFS, although only a weak benefit was observed (median PFS 5 months (95% CI: 3–11) vs. 6 months (95% CI: 5–13, *p* = 0.029) (Figure 1B). We also observed a strong beneficial impact of irAEs on the patients’ OS, as patients who experienced any irAEs had a significantly improved median OS of 37 months (95% CI: 24-NA) compared to 6 months (95% CI: 4–30) in patients without irAEs (*p* < 0.0001) (Figure 1C). When we further stratified by irAE grade, we detected only moderate differences, far less pronounced compared to the effect of irAE as such, between patients with grade 1–2 and grade ≥ 3 irAE (median OS 37 months (95% CI: 23-NA) vs. 33 months (95% CI: 23-NA), *p* < 0.0001).

**Figure 2:**
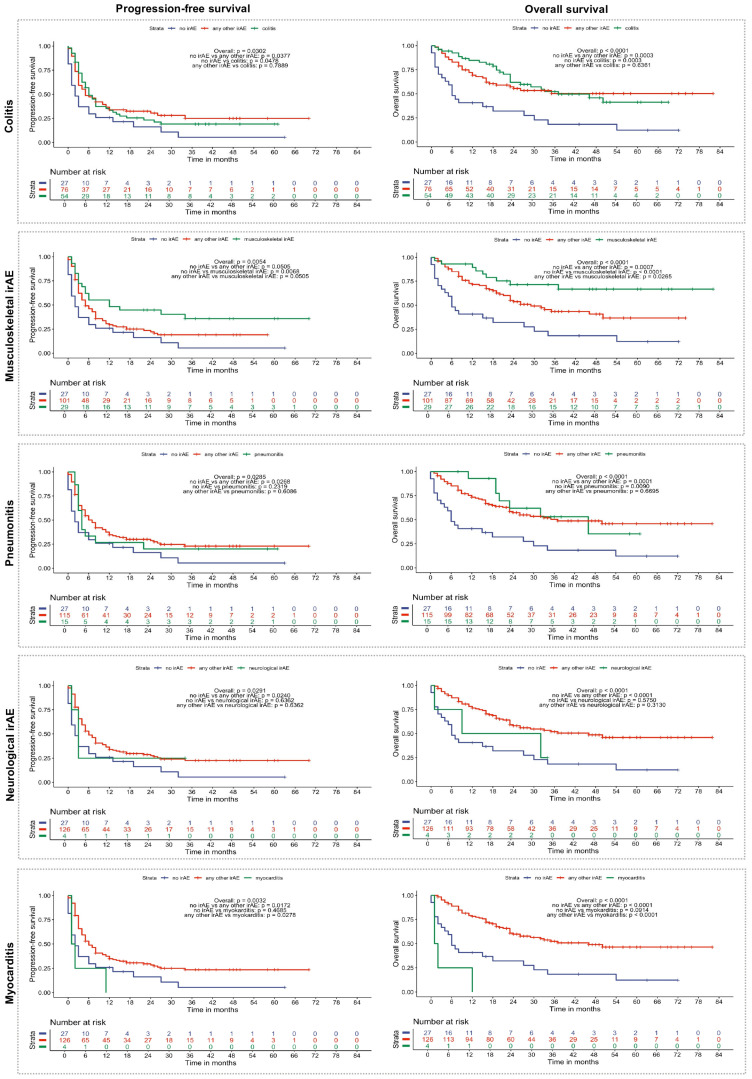

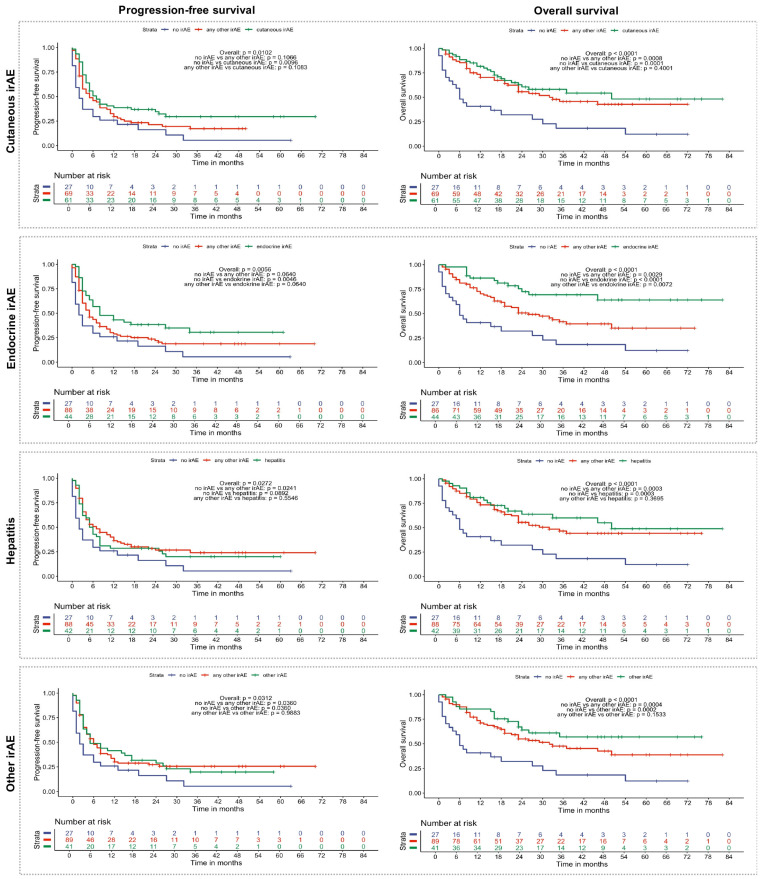
Progression-free and overall survival of melanoma patients depending on the subtype of irAE. PFS and OS were evaluated separately for different subtypes of irAE (for group definition see Section 2), comparing the respective subtype to patients without irAE and patients with any other irAE except for the respective irAE subtype. Overall *p*-value (log-rank test) and *p*-values of pairwise comparisons using the log-rank test with Benjamini–Hochberg correction are shown in the individual plots.

In the next step, we continued with a more detailed analysis of PFS and OS depending on the irAE subtype. For this purpose, we compared patients with a specific irAE subtype to patients with any other irAE and those without any irAE (Figure 2). We noticed that the beneficial effects of irAEs on PFS (Supplementary Table S6) and OS (Supplementary Table S7) clearly depended on the type of irAE. For example, patients with musculoskeletal irAEs showed a trend towards an improved median PFS of 12 months (95% CI: 5-NA) compared to 5 months (95% CI: 4–8) (*p* = 0.0505) in patients with any other irAE and a statistically significant higher PFS compared to patients without irAE (2 months, 95% CI: 1–8) (*p* = 0.0068). Interestingly, patients who experienced pneumonitis, neurological irAE, myocarditis, or hepatitis did not have a significantly improved PFS compared to patients who never experienced irAE during their treatment (*p* = 0.2319, *p* = 0.6362, *p* = 0.4685, and *p* = 0.089, respectively). Similar differences were observed regarding the OS, which was the best in patients with musculoskeletal irAEs (median OS not reached) compared to patients with other irAEs (29 months (95% CI: 23-NA)) (*p* = 0.0265) or no irAEs (6 months (95% CI: 4–30)) (*p* < 0.0001). Other irAEs, such as colitis or pneumonitis, were associated with significantly improved OS compared to the absence of irAEs (*p* = 0.0003 and *p* = 0.0090), but the effect was not significant compared to patients who experienced any other irAEs (*p* = 0.6361 and *p* = 0.6695). Neurological irAEs and myocarditis (both only *n* = 4) were rarely observed in our patient collective and should, therefore, be interpreted with caution, but both were associated with an impaired OS in contrast to any other irAEs. Patients with neurological irAEs had a reduced, but not statistically significant, median OS of 20.5 months (95% CI: 1-NA) compared to patients with any other irAEs (46 months, 95% CI: 24-NA, *p* = 0.3130) and compared to patients without irAEs (6 months, 95% CI: 4–30, *p* = 0.5750). In contrast, the median OS of patients affected by myocarditis (1.5 months, 95% CI: 1-NA) was below that of patients without any irAEs (6 months (95% CI: 4–30)) although not statistically significant (*p* = 0.0914) and statistically significant (*p* < 0.0001) below that of patients with any other irAEs (Figure 2).

A Cox proportional hazard analysis was conducted to identify additional factors associated with PFS (Table 6) and OS (Table 7) of melanoma patients during and after ICI. As already shown in Figure 1, the occurrence of irAEs was identified as a favorable factor for PFS in univariate analysis (HR 0.55, 95% CI: 0.35–0.86, *p* = 0.009), but was not significant in multivariate analysis (HR 0.61, 95% CI: 0.38–1.00, *p* = 0.051). Similarly, elevated LDH levels before therapy start were identified as a risk factor only in univariate but not multivariate analysis (univariate: HR 1.59, 95% CI: 1.07–1.79, *p* = 0.021, multivariate: HR 1.09, 95% CI: 9.67–1.77, *p* = 0.741). In contrast, in multivariate analysis, a high ECOG status of ≥2 (HR multivariate 3.41, 95% CI: 1.64–7.10, *p* = 0.001) and elevated S100B before the start of therapy (HR multivariate 2.17, 95% CI: 1.35–3.49, *p* = 0.001) were risk factors for disease progression. Additionally, patients with ocular melanoma had a higher risk of progression (HR multivariate: 2.93, 95% CI: 1.43–6.00, *p* = 0.003). No differences were observed between patients on monotherapy compared to combination therapy (univariate *p* = 0.301), as well as patients’ sex (univariate *p* = 0.992) and age (univariate *p* = 0.587) (Table 6).

With regard to OS, age at diagnosis was identified as a weak risk factor in univariate analysis (HR 1.02, 95% CI: 1.00–1.03, *p* = 0.013) but was not significant in multivariate analysis (*p* = 0.882). Similarly to PFS, an increased ECOG status of ≥2 (HR multivariate 5.02, 95% CI: 1.83–13.74, *p* = 0.002) was an independent risk factor for OS in multivariate analysis. In addition, ocular melanoma had the highest HR with respect to OS among the significant factors in multivariate analysis (HR multivariate 5.12, 95% CI: 1.91–13.74, *p* = 0.001). Elevated levels of S100B (HR univariate 1.95, 95% CI: 1.24–3.05, *p* = 0.004) and LDH (HR univariate 1.74, 95% CI: 1.05–2.88, *p* = 0.032) before the start of the therapy were identified as risk factors for OS in univariate analysis but did not sustain in multivariate analysis. In line with our survival time analysis, we identified the occurrence of irAE as a strong independent protective factor for OS in univariate, as well as multivariate analysis (HR multivariate 0.42, 95% CI: 0.21–0.81, *p* = 0.009) (Table 7).

**Figure 3:**
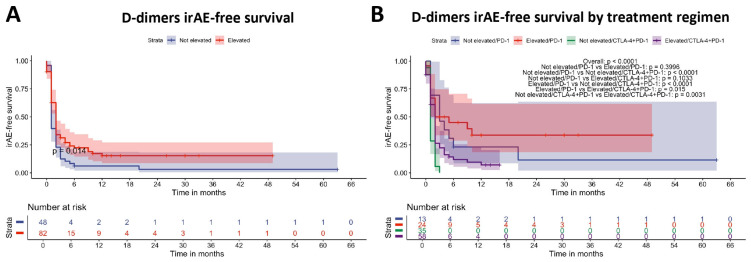
Association of the occurrence of irAE and the D-dimer level at baseline. (**A**) Time to first occurrence of irAE stratified by D-dimer level at therapy start. The overall log-rank *p*-value is shown. (**B**) Time to first occurrence of irAE stratified by D-dimer level at therapy start and the treatment regimen. Overall *p*-value (log-rank test) and *p*-values of pairwise comparisons using the log-rank test with Benjamini–Hochberg correction are shown.

As we could demonstrate that the occurrence of irAE is associated with disease-related outcomes, we further investigated the factors that may be used for the prediction of irAE and the irAE-free survival. Patients with elevated D-dimers had a longer median irAE-free survival of 1 month (2 months [95% CI: 2–2] vs. 1 month [95% CI: 1–2], *p* = 0.014) compared to patients with non-elevated D-dimers (Figure 3A). This finding persisted when stratified for treatment regimen (Figure 3B). ROC analysis resulted in a moderate discriminatory value of D-dimers for irAE with an area under the curve (AUC) of 0.711 (95% CI: 0.591–0.830) and an optimal cut-off (Youden) of 0.575 mg/L (specificity: 0.905, sensitivity: 0.468) (Supplementary Figure S1).

In the next step, Cox regression analysis was used to identify prognostic factors for irAE-free survival. In univariate analysis, ECOG 1 (HR: 0.6, [95% CI: 0.37–0.96], *p* = 0.035), PD-1 monotherapy (HR: 0.38, [95% CI: 0.28–0.58], *p* < 0.001) and elevated D-dimers (HR: 0.61 [95% CI: 0.42–0.90], *p* = 0.013) were significant beneficial factors for irAE-free survival. In multivariate Cox regression analysis, only PD-1 monotherapy (HR: 0.35 [95% CI: 0.21–0.56], *p* < 0.001) and elevated D-dimers (HR: 0.54, [95% CI: 0.36–0.81], *p* = 0.003) sustained as independent prognostic factors associated with a better irAE-free survival (Table 8).

## 4. Discussion

This study sheds light on the significance of the occurrence of different irAEs regarding prognosis and treatment response in the treatment of metastatic melanoma with ICI. D-dimers can serve as a predictive marker for irAE under ICI. The results provide new clinical insights into the dynamics between the activation of the immune and coagulation systems and the occurrence of irAEs and their influence on the clinical course.

In the study, more than 80% of patients suffered irAEs, which were often mild to moderate (CTCAE grade 1–2) in around 48% of those patients. However, severe to life-threatening (CTCAE grade 3–4) irAEs occurred in 52% of cases, particularly in patients receiving combination therapy with ipilimumab and nivolumab, which is in line with previous reports that combination regimens with CTLA-4 and PD-1 inhibitors are associated with higher toxicity [18,19].

D-dimers are a specific product of plasmin-degraded cross-linked fibrin and are elevated upon coagulatory and usually inflammatory activity [20]. In our work, elevated D-dimer levels prior to therapy initiation were identified as a protective factor for the occurrence of irAEs, which is surprising given the fact that D-dimers have been described as proinflammatory and would, therefore, most likely facilitate irAE occurrence. However, other markers for inflammation, such as CRP and immune cell subsets, were not significantly different in patients with irAE and without irAE, suggesting a complex relationship between coagulation, inflammation, and immune-related toxicity that we cannot deduce from the available data and should be investigated in future work.

Interestingly, our study showed that the severity of irAEs (mild vs. severe) had only a moderate impact on disease-related outcomes. These results emphasize that the occurrence of irAEs could be a sign of effective immune activation. The type of irAEs strongly influenced the prognosis. Musculoskeletal, endocrine, and cutaneous irAEs are associated with the longest PFS, whereas myocarditis, neurological irAEs, and pneumonitis are associated with the shortest PFS. With respect to OS, musculoskeletal irAE, endocrine irAE, and the group of other irAEs were associated with the longest OS, whereas patients experiencing myocarditis and neurological irAEs had the shortest OS.

The study provides evidence that the occurrence of irAEs could be a surrogate marker for the efficacy of ICIs. D-dimer levels could help stratify patients to identify those at higher risk of irAEs and those who have a shorter irAE-free survival time as an independent factor besides therapy regimen. Clinically, D-dimers are used as a marker to diagnose deep vein thrombosis or arterial pulmonary embolism. Also, it is known that D-dimer levels correlate with proinflammatory cytokine levels and predict outcomes in critically ill patients [21]. The influence of elevated D-dimers and the indicated coagulation activation [22,23] on the immune system is unclear. In this respect, more in-depth mechanistic studies are required. Moreover, recently it has been shown that D-dimer levels can predict ICI response in cutaneous squamous cell carcinoma patients treated with anti-PD-1 antibody cemiplimab, hence warranting further investigations on D-dimer treatment efficacy prediction in melanoma patients [24].

Other laboratory parameters that have been used in other studies as predictors of irAEs under ICI are AST, ALT, LDH, and absolute lymphocyte count in non-small cell lung cancer [25]. High disease burden, dual-agent ICI therapy, and low baseline platelet-to-lymphocyte ratio have been demonstrated to predict high-grade irAE [26]. Other predictive factors include gender, antibiotic use, post-treatment neutrophil-to-lymphocyte ratio, and baseline circulating tumor cell levels [27]. Machine learning models, using electronic patient-reported outcomes, can already predict the presence and onset of irAE [28].

Research suggests that specific immunological signatures correlate with different irAE subtypes [29]. Patients with severe irAEs showed a lower increase in regulatory effector T-cells and a higher Th17/Th1 ratio after anti-PD-1 treatment [29]. Different levels of certain proinflammatory markers were observed in patients who developed irAEs. In patients who developed irAEs, levels of CXCL9, CXCL10, CXCL11, and CXCL19 were lower at baseline, and after treatment, CXCL9 and CXCL10 levels increased more than in patients without irAEs [30]. In contrast, Alserawan et al. found a significant increase in IFN-induced cytokines such as CXCL9/10/11, IL-18, and IL-10 at the onset of the adverse event in patients with serious immune-related adverse events [31]. Elevated levels of IL-6 and CRP were observed in patients who developed irAEs from ICI treatment [32]. Self-antigens may play a role in mediating irAEs, particularly in cutaneous toxicity and pneumonitis [33]. A meta-analysis found that the occurrence of irAEs was significantly associated with better efficacy of ICI, especially for endocrine, dermatological, and low-grade irAEs [34]. This association was significant for PD-1 inhibitors, but not for CTLA-4 inhibitors [34]. Our analysis also shows that the type of irAEs plays an important role; well-fitting are the associations of improved response of ICI in cutaneous irAEs, which could indicate that certain immunological signatures correlate with specific irAEs. These findings should be further confirmed in larger, prospective studies, including melanoma patients treated with single-agent or combination immunotherapy (nivolumab or pembrolizumab versus ipilimumab plus nivolumab), which could contribute to the development of personalized therapy strategies in the future.

## 5. Conclusions

Despite the revealing results, our study also has certain limitations. The retrospective nature of the data collection limits the causal interpretation of the results. The focus on a single institution limits the generalizability of the results. In addition, a heterogeneous cohort, including uveal and mucosal melanoma, and small cohort sizes, for example, neurological irAEs and myocarditis or other rare to moderately common irAEs, limit the statistical power. Additionally, it should also be noted that due to the retrospective nature of the study, potential confounding factors, including the COVID-19 pandemic, could be present that may influence the analysis. Future studies could focus on prospective designs to validate the relationship between D-dimers, irAEs, and treatment success. In addition, the function of specific irAE subtypes in the immune response should be investigated in more depth to better understand the underlying mechanisms.

This study emphasizes the importance of D-dimers as a potential biomarker for the prediction of irAEs by ICI and highlights the prognostic significance of these irAEs in melanoma patients undergoing ICI. In the long term, the results could help to individualize the treatment of patients with ICIs and optimize the balance between toxicity and efficacy.

## Figures and Tables

**Table 1:** Overview of clinico-pathological characteristics of the melanoma patient population included in the study. ECOG: Eastern Cooperative Oncology Group; AJCC: American Joint Committee on Cancer; Response was encoded according to the recommendations from the Society for Immunotherapy of Cancer (SITC) [17]. *BRAF/NRAS/cKIT* mutational status based on routine tissue analysis. * As patients could present with multiple metastatic sites and had multiple previous treatments, the column percentages of the total N exceed 100% for these variables.

	Total N	Missing N		N (%)
**Sex**	157	0	Female	55 (35.0)
			Male	102 (65.0)
**Age at start of therapy in years**	157	0	Mean (SD)	66.0 (15.9)
**ECOG**	157	0	0	117 (74.5)
			1	29 (18.5)
			≥2	11 (7.0)
**Histology type**	157	0	Superficial spreading melanoma	29 (18.5)
			Nodular melanoma	27 (17.2)
			Acrolentiginous melanoma	3 (1.9)
			Ocular melanoma	16 (10.2)
			Mucosal melanoma	18 (11.5)
			Cancer of unknown primary	30 (19.1)
			Other cutaneous melanomas	34 (21.7)
**AJCC stage**	157	0	III	28 (17.8)
			IV	129 (82.2)
Metastatic sites *	121	0		
			Cutaneous	6 (5.0) *
			Soft tissue	29 (24.0) *
			Distant lymph nodes	50 (41.3) *
			Brain	32 (26.4) *
			Liver	38 (31.4) *
			Lung	78 (64.5) *
			Bone	17 (14.0) *
			Other visceral metastasis	28 (23.1) *
**Therapy regimen at inclusion**	157	0	CTLA-4 + PD-1	111 (70.7)
			PD-1	46 (29.3)
**Therapy line**	157	0	first line	116 (73.9)
			second line	33 (21.0)
			third line	6 (3.8)
			fourth line	2 (1.3)
Previous therapy regimens *	41	0	Interferon	8 (19.5) *
			BRAF/MEK inhibitors	12 (29.3) *
			PD-1	31 (75.6) *
**Response**	157	0	Response	37 (23.6)
			Primary Resistance	91 (58.0)
			Secondary Resistance	29 (18.5)
***BRAF*** **mutational status**	139	18	wild type	86 (61.9)
			mutated	53 (38.1)
***NRAS*** **mutational status**	128	29	wild type	94 (73.4)
			mutated	34 (26.6)
***cKIT*** **mutational status**	124	33	wild type	121 (97.6)
			mutated	3 (2.4)
**Ever experienced irAE**	157	0	No irAE	27 (17.2)
			irAE	130 (82.8)

**Table 2:** Descriptive table of irAE observed during ICI treatment of melanoma patients (grading according to CTCAE).

	Total N		N (%)
**Ever experienced irAE**	157	No irAE	27 (17.2)
		irAE	130 (82.8)
**First irAE grade**	130	Grade 1–2	93 (71.5)
		Grade ≥ 3	37 (28.5)
**Strongest irAE grade**	130	Grade 1–2	62 (47.7)
		Grade ≥ 3	68 (52.3)
**Total number of irAEs**	130	1	65 (50.0)
		2	25 (19.2)
		3	23 (17.7)
		4	12 (9.2)
		5	4 (3.1)
		6	1 (0.8)

**Table 3:** Spectrum of irAE observed during ICI treatment of melanoma patients (grading according to CTCAE).

	Total N		N (%)
**Cutaneous irAE**	61	Unknown, but not hospitalized	12 (19.7)
		Grade 1–2	45 (73.7)
		Grade ≥ 3	4 (6.6)
**Colitis**	54	Grade 1–2	19 (35.2)
		Grade ≥ 3	35 (64.8)
**Hepatitis**	42	Grade 1–2	19 (45.2)
		Grade ≥ 3	23 (54.8)
**Endocrine irAE**	44	Unknown, but not hospitalized	4 (9.1)
		Grade 1–2	31 (70.4)
		Grade ≥ 3	9 (20.5)
**Musculoskeletal irAE**	29	Unknown, but not hospitalized	1 (3.4)
		Grade 1–2	24 (82.8)
		Grade ≥ 3	4 (13.8)
**Neurological irAE**	4	Unknown, but not hospitalized	2 (50.0)
		Grade 1–2	1 (25.0)
		Grade ≥ 3	1 (25.0)
**Myocarditis**	4	Grade 1–2	1 (25.0)
		Grade ≥ 3	3 (75.0)
**Pneumonitis**	15	Grade 1–2	10 (66.7)
		Grade ≥ 3	5 (33.3)
**Other irAE**	41	Unknown, but not hospitalized	19 (46.3)
		Grade 1–2	13 (31.7)
		Grade ≥ 3	9 (22.0)

**Table 4:** Clinico-pathological parameters grouped by the occurrence of any irAEs. ECOG: Eastern Cooperative Oncology Group; AJCC: American Joint Committee on Cancer; Response was encoded according to the recommendations from the Society for Immunotherapy of Cancer (SITC) [17]. *BRAF/NRAS/cKIT* mutational status based on routine tissue analysis. (a) Fisher’s exact test (b) two-sided *t* test.

	Total N		No irAE N (%)	irAE N (%)	*p* Value
**Sex**	157	Female	11 (40.7)	44 (33.8)	0.512 (a)
		Male	16 (59.3)	86 (66.2)	
**Age at start of therapy in years**	157	Mean (SD)	71.3 (19.4)	64.9 (14.9)	0.059 (b)
**ECOG**	157	0	13 (48.1)	104 (80.0)	**0.002** (a)
		1	9 (33.3)	20 (15.4)	
		≥2	5 (18.5)	6 (4.6)	
**Histology type**	157	Superficial spreading melanoma	5 (18.5)	24 (18.5)	0.945 (a)
		Nodular melanoma	4 (14.8)	23 (17.5)	
		Acrolentiginous melanoma	1 (3.7)	2 (1.5)	
		Ocular melanoma	3 (11.1)	13 (10.0)	
		Mucosal melanoma	2 (7.4)	16 (12.3)	
		Cancer of unknown primary	6 (22.2)	24 (18.5)	
		Other cutaneous melanomas	6 (22.2)	28 (21.5)	
**AJCC stage**	157	III	5 (18.5)	23 (17.7)	1.000 (a)
		IV	22 (81.5)	107 (82.3)	
**Therapy**	157	CTLA-4 + PD-1	11 (40.7)	100 (76.9)	**<0.001** (a)
		PD-1	16 (59.3)	30 (23.1)	
**Therapy line**	157	first line	21 (77.8)	95 (73.1)	0.925 (a)
		second line	5 (18.5)	28 (21.5)	
		≥third line	1 (3.7)	7 (5.4)	
**Response**	157	Response	3 (11.1)	34 (26.2)	0.223 (a)
		Primary Resistance	19 (70.4)	72 (55.4)	
		Secondary Resistance	5 (18.5)	24 (18.5)	
***BRAF*** **mutational status**	139	wild type	15 (57.7)	71 (62.8)	0.659 (a)
		mutated	11 (42.3)	42 (37.2)	
***NRAS*** **mutational status**	128	wild type	19 (76.0)	75 (72.8)	1.000 (a)
		mutated	6 (24.0)	28 (27.2)	
***cKIT*** **mutational status**	124	wild type	22 (95.7)	99 (98.0)	0.463 (a)
		mutated	1 (4.3)	2 (2.0)	

**Table 5:** Laboratory parameters at baseline grouped by the occurrence of irAEs. NLR: neutrophil-to-lymphocyte ratio; IQR: interquartile range. *p*-values were calculated using the Kruskal–Wallis test.

	Total N		No irAE	irAE	*p* Value
**LDH U/L**	154	Median (IQR)	343.00 (261.00 to 684.00)	271.00 (229.00 to 355.00)	**0.025**
**S100B µg/L**	152	Median (IQR)	0.33 (0.13 to 0.75)	0.13 (0.07 to 0.54)	**0.031**
**D-dimers mg/L**	130	Median (IQR)	1.29 (0.81 to 3.37)	0.60 (0.37 to 1.34)	**0.002**
**CRP mg/L**	151	Median (IQR)	2.00 (2.00 to 63.00)	3.00 (2.00 to 20.25)	0.529
**Leukocytes × 10^9^/L**	154	Median (IQR)	7.65 (6.40 to 9.02)	6.90 (5.80 to 8.43)	0.164
**Neutrophils × 10^9^/L**	154	Median (IQR)	4.86 (4.23 to 7.16)	4.65 (3.61 to 5.54)	0.290
**Lymphocytes × 10^9^/L**	154	Median (IQR)	1.19 (1.01 to 1.76)	1.52 (1.19 to 1.88)	0.113
**NLR**	154	Median (IQR)	3.72 (2.15 to 7.08)	3.08 (2.29 to 4.22)	0.246

**Table 6:** Univariable and multivariable Cox proportional hazard analysis of progression-free survival in melanoma patients under ICI treatment. HR: Hazard ratio; CI: confidence interval; SD: standard deviation; ECOG: Eastern Cooperative Oncology Group; AJCC: American Joint Committee on Cancer.

Progression-Free Survival		N (%)	HR (Univariable) (95% CI, *p* Value)	HR (Multivariable) (95% CI, *p* Value)
**Sex**	Female	55 (35.0)	-	-
	Male	102 (65.0)	1.00 (0.69–1.46, *p* = 0.992)	-
**Age at diagnosis in years**	Mean (SD)	63.2 (16.4)	1.00 (0.99–1.01, *p* = 0.587)	-
**ECOG**	0	117 (74.5)	-	-
	1	29 (18.5)	1.15 (0.73–1.83, *p* = 0.541)	0.91 (0.53–1.56, *p* = 0.726)
	≥2	11 (7.0)	**3.10 (1.59–6.02, *p* = 0.001)**	**3.41 (1.64–7.10, *p* = 0.001)**
**Histology type**	Superficial spreading melanoma	29 (18.5)	-	-
	Nodular melanoma	27 (17.2)	1.33 (0.72–2.48, *p* = 0.363)	1.16 (0.62–2.18, *p* = 0.635)
	Acrolentiginous melanoma	3 (1.9)	2.07 (0.61–6.99, *p* = 0.244)	1.22 (0.36–4.22, *p* = 0.748)
	Ocular melanoma	16 (10.2)	**2.18 (1.10–4.29, *p* = 0.025)**	**2.93 (1.43–6.00, *p* = 0.003)**
	Mucosal melanoma	18 (11.5)	1.59 (0.81–3.13, *p* = 0.182)	1.99 (0.96–4.12, *p* = 0.065)
	Cancer of unknown primary	30 (19.1)	1.23 (0.67–2.25, *p* = 0.512)	1.02 (0.55–1.89, *p* = 0.961)
	Other cutaneous melanomas	34 (21.7)	1.09 (0.60–1.99, *p* = 0.780)	1.17 (0.62–2.23, *p* = 0.622)
**AJCC stage**	III	28 (17.8)	-	-
	IV	129 (82.2)	1.57 (0.95–2.60, *p* = 0.079)	-
**Therapy**	CTLA-4 + PD-1	111 (70.7)	-	-
	PD-1	46 (29.3)	1.22 (0.84–1.79, *p* = 0.301)	-
**LDH U/L**	Not elevated	50 (32.5)	-	-
	Elevated	104 (67.5)	**1.59 (1.07–2.37, *p* = 0.021)**	1.09 (0.67–1.77, *p* = 0.741)
**S100B µg/L**	Not elevated	76 (50.0)	-	-
	Elevated	76 (50.0)	**1.99 (1.38–2.87, *p* < 0.001)**	**2.17 (1.35–3.49, *p* = 0.001)**
**Adverse events**	No irAE	27 (17.2)	-	-
	irAE	130 (82.8)	**0.55 (0.35–0.86, *p* = 0.009)**	0.61 (0.38–1.00, *p* = 0.051)

**Table 7:** Univariable and multivariable Cox proportional hazard analysis of overall survival in melanoma patients under ICI treatment. HR: hazard ratio; CI: confidence interval; SD: standard deviation; ECOG: Eastern Cooperative Oncology Group; AJCC: American Joint Committee on Cancer.

Overall Survival		N (%)	HR (Univariable) (95% CI, *p* Value)	HR (Multivariable) (95% CI, *p* Value)
**Sex**	Female	55 (35.0)	-	-
	Male	102 (65.0)	1.07 (0.68–1.70, *p* = 0.763)	-
**Age at diagnosis in years**	Mean (SD)	63.2 (16.4)	**1.02 (1.00–1.03, *p* = 0.013)**	1.00 (0.98–1.02, *p* = 0.882)
**ECOG**	0	117 (74.5)	-	-
	1	29 (18.5)	**2.21 (1.32–3.71, *p* = 0.003)**	1.74 (0.78–3.90, *p* = 0.176)
	≥2	11 (7.0)	**8.07 (3.96–16.41, *p* < 0.001)**	**5.02 (1.83–13.74, *p* = 0.002)**
**Histology type**	Superficial spreading melanoma	29 (18.5)	-	-
	Nodular melanoma	27 (17.2)	1.23 (0.56–2.70, *p* = 0.606)	1.19 (0.47–3.01, *p* = 0.717)
	Acrolentiginous melanoma	3 (1.9)	2.40 (0.53–10.76, *p* = 0.253)	0.00 (0.00-Inf, *p* = 0.996)
	Ocular melanoma	16 (10.2)	**2.78 (1.24–6.20, *p* = 0.013)**	**5.12 (1.91–13.74, *p* = 0.001)**
	Mucosal melanoma	18 (11.5)	1.66 (0.73–3.76, *p* = 0.226)	1.61 (0.55–4.73, *p* = 0.386)
	Cancer of unknown primary	30 (19.1)	1.10 (0.52–2.36, *p* = 0.797)	1.04 (0.42–2.58, *p* = 0.939)
	Other cutaneous melanomas	34 (21.7)	1.26 (0.60–2.63, *p* = 0.547)	1.08 (0.42–2.78, *p* = 0.881)
**AJCC stage**	III	28 (17.8)	-	-
	IV	129 (82.2)	1.21 (0.67–2.19, *p* = 0.532)	-
**Therapy**	CTLA-4 + PD-1	111 (70.7)	-	-
	PD-1	46 (29.3)	1.14 (0.72–1.79, *p* = 0.586)	-
**LDH U/L**	Not elevated	50 (32.5)	-	-
	Elevated	104 (67.5)	**1.74 (1.05–2.88, *p* = 0.032)**	1.24 (0.61–2.49, *p* = 0.555)
**S100B µg/L**	Not elevated	76 (50.0)	-	-
	Elevated	76 (50.0)	**1.95 (1.24–3.05, *p* = 0.004)**	1.34 (0.69–2.62, *p* = 0.385)
**Adverse events**	No irAE	27 (17.2)	-	-
	irAE	130 (82.8)	**0.35 (0.21–0.57, *p* < 0.001)**	**0.42 (0.21–0.81, *p* = 0.009)**

**Table 8:** Univariable and multivariable Cox proportional hazard analysis of time to first occurrence of irAE in melanoma patients under ICI treatment. HR: Hazard ratio; CI: confidence interval; SD: standard deviation; ECOG: Eastern Cooperative Oncology Group; AJCC: American Joint Committee on Cancer; T: tumor, N: nodes, M: metastases (according to the TNM classification).

irAE-Free Survival		N (%)/Mean (SD)	HR (Univariable) (95% CI, *p* Value)	HR (Multivariable) (95% CI, *p* Value)
**Sex**	Female	55 (35.0)	-	-
	Male	102 (65.0)	1.10 (0.76–1.58, *p* = 0.624)	-
**Age at diagnosis in years**	Mean (SD)	63.2 (16.4)	0.99 (0.98–1.00, *p* = 0.117)	-
**ECOG**	0	117 (74.5)	-	-
	1	29 (18.5)	**0.60 (0.37–0.96, *p* = 0.035)**	0.65 (0.38–1.12, *p* = 0.118)
	≥2	11 (7.0)	0.74 (0.33–1.70, *p* = 0.484)	1.86 (0.72–4.80, *p* = 0.199)
**Histology type**	Superficial spreading melanoma	29 (18.5)	-	-
	Nodular melanoma	27 (17.2)	0.98 (0.55–1.73, *p* = 0.937)	-
	Acrolentiginous melanoma	3 (1.9)	0.59 (0.14–2.50, *p* = 0.475)	-
	Ocular melanoma	16 (10.2)	1.42 (0.72–2.81, *p* = 0.317)	-
	Mucosal melanoma	18 (11.5)	0.99 (0.53–1.87, *p* = 0.984)	-
	Cancer of unknown primary	30 (19.1)	1.00 (0.57–1.76, *p* = 0.993)	-
	Other cutaneous melanomas	34 (21.7)	0.95 (0.55–1.64, *p* = 0.855)	-
**AJCC stage**	III	28 (17.8)	-	-
	IV	129 (82.2)	1.21 (0.77–1.90, *p* = 0.414)	-
**Therapy line**	First line	116 (73.9)	-	-
	Second line	33 (21.0)	1.18 (0.77–1.80, *p* = 0.440)	-
	≥Third line	8 (5.1)	0.89 (0.41–1.91, *p* = 0.757)	-
**Baseline therapy**	CTLA-4 + PD-1	111 (70.7)	-	-
	PD-1	46 (29.3)	**0.38 (0.25–0.58, *p* < 0.001)**	**0.35 (0.21–0.56, *p* < 0.001)**
**LDH U/L**	Not elevated	50 (32.5)	-	-
	Elevated	104 (67.5)	0.88 (0.61–1.27, *p* = 0.507)	-
**S100B µg/L**	Not elevated	76 (50.0)	-	-
	Elevated	76 (50.0)	0.76 (0.53–1.08, *p* = 0.121)	-
**D-dimers mg/L**	Not elevated	48 (36.9)	-	-
	Elevated	82 (63.1)	**0.61 (0.42–0.90, *p* = 0.013)**	**0.54 (0.36–0.81, *p* = 0.003)**
**CRP mg/L**	Mean (SD)	23.1 (47.4)	1.00 (0.99–1.00, *p* = 0.652)	-
**Leukocytes × 10^9^/L**	Mean (SD)	7.7 (3.2)	0.98 (0.93–1.05, *p* = 0.609)	-
**Neutrophils × 10^9^/L**	Mean (SD)	5.4 (3.6)	0.97 (0.93–1.02, *p* = 0.305)	-
**Lymphocytes × 10^9^/L**	Mean (SD)	1.7 (1.6)	1.06 (0.97–1.17, *p* = 0.194)	-
**NLR**	Mean (SD)	4.3 (5.3)	0.96 (0.92–1.01, *p* = 0.091)	-

## Data Availability

The data that support the findings of this study are available from the corresponding authors (D.J.S. and C.G.) upon reasonable request.

## Supplementary Materials

The following supporting information can be downloaded at: https://www.mdpi.com/article/10.3390/cancers17172806/s1, Supplementary Figure S1: ROC analysis D-dimer status to predict irAE occurrence; Supplementary Table S1: Specification of irAE subtype and corresponding grades observed in the study population; Supplementary Table S2: Spectrum of irAE observed during ICI treatment of melanoma patients stratified by sex; Supplementary Table S3: Spectrum of irAE observed during ICI treatment of melanoma patients stratified by major subtypes. *p* values were calculated using Fisher’s exact test; Supplementary Table S4: Clinico-pathological parameters grouped by the grade of the strongest irAE; Supplementary Table S5: Laboratory serum parameters at baseline grouped by the grade of the strongest irAE; Supplementary Table S6: Tabular overview of median PFS outcomes as shown in Figure 2; Supplementary Table S7: Tabular overview of median OS outcomes as shown in Figure 2.

## Abbreviation

The following abbreviations are used in this manuscript:

AJCC	American Joint Committee on Cancer
ALT	alanine aminotransferase
AST	aspartate aminotransferase
CI	confidence interval
CRP	C-reactive protein
CTCAE	Common Terminology Criteria for Adverse Events
CTLA-4	cytotoxic T-lymphocyte-associated protein-4
CXCL	chemokine (C-X-C motif) ligand
ECOG	Eastern Cooperative Oncology
HR	hazard ratio
ICI	immune checkpoint inhibition
IQR	interquartile range
irAE	immune-related adverse events
LDH	lactate dehydrogenase
LoD	limit of detection
NA	Not applicable
NLR	neutrophil-to-lymphocyte ratio
OS	overall survival
PD	progressive disease
PD-1	programmed cell death protein-1
PFS	progression-free survival
RECIST	Response Evaluation Criteria in Solid Tumors
SD	standard deviation
SITC	Society for Immunotherapy of Cancer
Th	T-helper lymphocytes
TNF-α	tumor necrosis factor alpha
