# Supplementary material for: Comprehensive Assessment of Prognostic Factors for Immune-Related Adverse Events in Immune Checkpoint Inhibitor-Treated Melanoma
Source: Cancers (Basel). 2025 Aug 27;17(17):2806. doi: 10.3390/cancers17172806 (PMC12427384; doi:10.3390/cancers17172806)
Supplement: Supplementary file 1 [file cancers-17-02806-s001.zip › cancers-3761164-supplementary.pdf]

cancers

Supplementary Materials

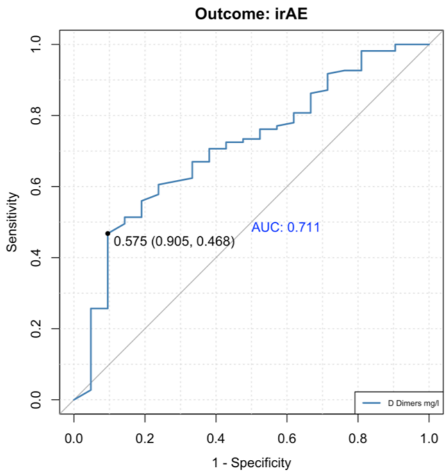

Supplementary Figure S1. ROC analysis D-dimer status to predict irAE occurrence. ROC analysis of the D-dimer level at baseline for the prediction of the occurrence of irAE, the optimal cut off (Youden) and respective values for 1-specificity and sensitivity at the optimal cut-off point are indicated on the plot; AUC: area under the curve.

Supplementary Table S1. Specification of irAE subtype and corresponding grades observed in the study population.

	Total N	Grade	N (%)
**Grade of first irAE**	130	Unknown, but not hospitalized	22 (16.9)
		1	32 (24.6)
		2	39 (30.0)
		3	30 (23.1)
		4	7 (5.4)
**Grade of strongest irAE**	130	Unknown, but not hospitalized	13 (10.0)
		1	16 (12.3)
		2	33 (25.4)
		3	47 (36.2)
		4	20 (15.4)
		5	1 (0.8)
**Colitis**	54	Unknown, but not hospitalized	1 (1.9)
		1	7 (13.0)
		2	11 (20.4)
		3	27 (50.0)
		4	8 (14.8)
**Hepatitis**	42	Unknown, but not hospitalized	11 (26.2)
		1	1 (2.4)
		2	7 (16.7)
		3	15 (35.7)
		4	8 (19.0)
**Gastritis**	6	1	2 (33.3)
		2	2 (33.3)
		3	2 (33.3)
**Nephritis**	8	Unknown, but not hospitalized	3 (37.5)
		2	1 (12.5)
		3	3 (37.5)
		4	1 (12.5)

	Total N	Grade	N (%)
**Thyroiditis**	28	1	7 (25.0)
		2	19 (67.9)
		3	2 (7.1)
**Pancreatitis**	8	Unknown, but not hospitalized	1 (12.5)
		1	5 (62.5)
		2	1 (12.5)
		4	1 (12.5)
**Diabetes mellitus**	4	Unknown, but not hospitalized	3 (75.0)
		3	1 (25.0)
**Hypophysitis**	12	Unknown, but not hospitalized	3 (25.0)
		2	2 (16.7)
		3	7 (58.3)
**Dermatitis**	51	Unknown, but not hospitalized	3 (5.9)
		1	36 (70.6)
		2	8 (15.7)
		3	3 (5.9)
		4	1 (2.0)
**Lichen ruber**	2	Unknown, but not hospitalized	1 (50.0)
		1	1 (50.0)
**Vitiligo**	11	Unknown, but not hospitalized	11 (100.0)
**Alopecia**	1	Unknown, but not hospitalized	1 (100.0)
**Pneumonitis**	15	Unknown, but not hospitalized	1 (6.7)
		1	5 (33.3)
		2	4 (26.7)
		3	4 (26.7)
		4	1 (6.7)
**Myocarditis**	4	2	1 (25.0)
		3	1 (25.0)
		4	2 (50.0)
**Neuritis**	1	Unknown, but not hospitalized	1 (100.0)
**Myelitis**	1	2	1 (100.0)
**Myasthenia**	2	Unknown, but not hospitalized	1 (50.0)
		5	1 (50.0)
**Sarcoid-like reaction**	8	Unknown, but not hospitalized	4 (50.0)
		1	4 (50.0)
**Arthritis**	21	Unknown, but not hospitalized	1 (4.8)
		1	5 (23.8)
		2	14 (66.7)
		3	1 (4.8)
**Myositis**	9	1	5 (55.6)
		2	1 (11.1)
		3	2 (22.2)
		4	1 (11.1)
**Anemia**	8	Unknown, but not hospitalized	5 (62.5)
		3	3 (37.5)
**Lymphopenia**	1	Unknown, but not hospitalized	1 (100.0)
**Fatigue**	5	Unknown, but not hospitalized	4 (80.0)
		2	1 (20.0)
**Infusion reaction**	4	Unknown, but not hospitalized	3 (75.0)
		2	1 (25.0)
**Uveitis**	1	Unknown, but not hospitalized	1 (100.0)
**Panniculitis**	1	2	1 (100.0)
**Sialadenitis**	1	Unknown, but not hospitalized	1 (100.0)
**Mucositis**	1	3	1 (100.0)
**Vasculitis**	2	Unknown, but not hospitalized	1 (50.0)
		1	1 (50.0)
**Sjörgen-like syndrome**	1	1	1 (100.0)

Supplementary Table S2. Spectrum of irAE observed during ICI treatment of melanoma patients stratified by sex. *p* values were calculated using the Fisher's exact test.

	Total N		Female N (%)	Male N (%)	*p* value
**Ever experienced irAE**	157	No irAE	11 (20.0)	16 (15.7)	0.512
		irAE	44 (80.0)	86 (84.3)	
**Cutaneous irAE**	157	No	37 (67.3)	59 (57.8)	0.304
		Yes	18 (32.7)	43 (42.2)	
**Colitis**	157	No	36 (65.5)	67 (65.7)	1.000
		Yes	19 (34.5)	35 (34.3)	
**Hepatitis**	157	No	34 (61.8)	81 (79.4)	**0.023**
		Yes	21 (38.2)	21 (20.6)	
**Endocrine irAE**	157	No	36 (65.5)	77 (75.5)	0.196
		Yes	19 (34.5)	25 (24.5)	
**Musculoskeletal irAE**	157	No	43 (78.2)	85 (83.3)	0.518
		Yes	12 (21.8)	17 (16.7)	
**Neurological irAE**	157	No	53 (96.4)	100 (98.0)	0.612
		Yes	2 (3.6)	2 (2.0)	
**Myocarditis**	157	No	53 (96.4)	100 (98.0)	0.612
		Yes	2 (3.6)	2 (2.0)	
**Pneumonitis**	157	No	50 (90.9)	92 (90.2)	1.000
		Yes	5 (9.1)	10 (9.8)	
**Other irAE**	157	No	43 (78.2)	73 (71.6)	0.448
		Yes	12 (21.8)	29 (28.4)	

Supplementary Table S3. Spectrum of irAE observed during ICI treatment of melanoma patients stratified by major subtypes. *p* values were calculated using the Fisher's exact test.

	Total N		CUP N (%)	Cutaneous N (%)	Mucosal N (%)	Uveal N (%)	*p* value
**Ever experienced irAE**	157	No irAE	6 (20.0)	16 (17.2)	2 (11.1)	3 (18.8)	0.895
		irAE	24 (80.0)	77 (82.8)	16 (88.9)	13 (81.2)	
**Cutaneous irAE**	157	No	17 (56.7)	61 (65.6)	11 (61.1)	7 (43.8)	0.378
		Yes	13 (43.3)	32 (34.4)	7 (38.9)	9 (56.2)	
**Colitis**	157	No	17 (56.7)	62 (66.7)	12 (66.7)	12 (75.0)	0.645
		Yes	13 (43.3)	31 (33.3)	6 (33.3)	4 (25.0)	
**Hepatitis**	157	No	22 (73.3)	68 (73.1)	11 (61.1)	14 (87.5)	0.407
		Yes	8 (26.7)	25 (26.9)	7 (38.9)	2 (12.5)	
**Endocrine irAE**	157	No	21 (70.0)	64 (68.8)	13 (72.2)	15 (93.8)	0.211
		Yes	9 (30.0)	29 (31.2)	5 (27.8)	1 (6.2)	
**Musculoskeletal irAE**	157	No	23 (76.7)	76 (81.7)	15 (83.3)	14 (87.5)	0.851
		Yes	7 (23.3)	17 (18.3)	3 (16.7)	2 (12.5)	

**Neurological irAE**	157	No	29 (96.7)	92 (98.9)	16 (88.9)	16 (100.0)	0.096
		Yes	1 (3.3)	1 (1.1)	2 (11.1)	0 (0.0)	
**Myocarditis**	157	No	30 (100.0)	91 (97.8)	17 (94.4)	15 (93.8)	0.284
		Yes	0 (0.0)	2 (2.2)	1 (5.6)	1 (6.2)	
**Pneumonitis**	157	No	23 (76.7)	88 (94.6)	16 (88.9)	15 (93.8)	**0.034**
		Yes	7 (23.3)	5 (5.4)	2 (11.1)	1 (6.2)	
**Other irAE**	157	No	23 (76.7)	70 (75.3)	13 (72.2)	10 (62.5)	0.692
		Yes	7 (23.3)	23 (24.7)	5 (27.8)	6 (37.5)	

Supplementary Table S4. Clinico-pathological parameters grouped by the grade of the strongest irAE. ECOG: Eastern Cooperative Oncology Group; AJCC: American Joint Committee on Cancer; T: tumor, N: nodes, M: metastases (according to the TNM classification); *BRAF*/*NRAS*/*cKIT* mutational status based on routine tissue analysis. (a) Fisher's exact test (b) one-way ANOVA.

	Total N		no irAE N (%)	Grade 1-2 N (%)	Grade ≥3 N (%)	*p* value
**Sex**	157	Female	11 (40.7)	20 (32.3)	24 (35.3)	0.741 (a)
		Male	16 (59.3)	42 (67.7)	44 (64.7)	
**Age at start of therapy in years**	157	Mean (SD)	71.3 (19.4)	65.1 (16.0)	64.7 (14.0)	0.167 (b)
**ECOG**	157	0	13 (48.1)	50 (80.6)	54 (79.4)	**0.010** (a)
		1	9 (33.3)	9 (14.5)	11 (16.2)	
		≥2	5 (18.5)	3 (4.8)	3 (4.4)	
**Histology type**	135	Superficial spreading melanoma	5 (18.5)	10 (16.1)	14 (20.6)	0.989 (a)
		Nodular melanoma	4 (14.8)	9 (14.5)	14 (20.6)	
		Acrolentiginous melanoma	1 (3.7)	1 (1.6)	1 (1.5)	
		Ocular melanoma	3 (11.1)	7 (11.3)	6 (8.8)	
		Mucosal melanoma	2 (7.4)	8 (12.9)	8 (11.8)	
		Cancer of unknown primary	6 (22.2)	11 (17.7)	13 (19.1)	
		Other cutaneous melanomas	6 (22.2)	16 (25.8)	12 (17.6)	
**AJCC stage**	157	III	5 (18.5)	10 (16.1)	13 (19.1)	0.901 (a)
		IV	22 (81.5)	52 (83.9)	55 (80.9)	
**Baseline therapy**	157	CTLA-4 + PD-1	11 (40.7)	41 (66.1)	59 (86.8)	**<0.001** (a)
		PD-1	16 (59.3)	21 (33.9)	9 (13.2)	
**Therapy line**	157	first line	21 (77.8)	47 (75.8)	48 (70.6)	0.829 (a)
		second line	5 (18.5)	11 (17.7)	17 (25.0)	
		≥ third line	1 (3.7)	4 (6.5)	3 (4.4)	
**Response**	157	Response	3 (11.1)	16 (25.8)	18 (26.5)	0.476 (a)
		Primary Resistance	19 (70.4)	36 (58.1)	36 (52.9)	
		Secondary Resistance	5 (18.5)	10 (16.1)	14 (20.6)	
***BRAF* mutational status**	139	wild type	15 (57.7)	29 (58.0)	42 (66.7)	0.570 (a)
		mutated	11 (42.3)	21 (42.0)	21 (33.3)	
***NRAS* mutational status**	128	wild type	19 (76.0)	34 (73.9)	41 (71.9)	0.925 (a)
		mutated	6 (24.0)	12 (26.1)	16 (28.1)	
***cKIT* mutational status**	124	wild type	22 (95.7)	43 (97.7)	56 (98.2)	0.789 (a)
		mutated	1 (4.3)	1 (2.3)	1 (1.8)	

Supplementary Table S5. Laboratory serum parameters at baseline grouped by the grade of the strongest irAE. NLR: neutrophil-to-lymphocyte ratio; IQR: interquartile range. *p* values were calculated using the Kruskal-Wallis test.

	Total N		no irAE	Grade 1-2	Grade ≥3	*p* value
**LDH U/L**	154	Median (IQR)	343.00 (261.00 to 684.00)	271.00 (236.75 to 366.25)	265.00 (228.00 to 336.50)	0.069
**S100B µg/L**	152	Median (IQR)	0.33 (0.13 to 0.75)	0.16 (0.08 to 0.46)	0.12 (0.06 to 0.61)	0.092
**D-dimers mg/L**	130	Median (IQR)	1.29 (0.81 to 3.37)	0.58 (0.41 to 1.21)	0.63 (0.35 to 1.35)	**0.009**
**CRP mg/L**	151	Median (IQR)	2.00 (2.00 to 63.00)	2.00 (2.00 to 19.50)	5.00 (2.00 to 19.00)	0.759
**Leukocytes x10⁹/L**	154	Median (IQR)	7.65 (6.40 to 9.02)	7.10 (5.80 to 8.50)	6.70 (5.90 to 8.20)	0.338
**Neutrophils x10⁹/L**	154	Median (IQR)	4.86 (4.23 to 7.16)	4.80 (3.60 to 5.56)	4.55 (3.65 to 5.45)	0.553
**Lymphocytes x10⁹/L**	154	Median (IQR)	1.19 (1.01 to 1.76)	1.48 (1.11 to 1.82)	1.53 (1.27 to 1.89)	0.204
**NLR**	154	Median (IQR)	3.72 (2.15 to 7.08)	3.19 (2.34 to 4.52)	2.96 (2.27 to 3.89)	0.454

Supplementary Table S6. Tabular overview of median PFS outcomes as shown in Figure 2. Overall *p* values were calculated using the log-rank test.

PFS		N	Median PFS in months (95% CI, *p* value)	*p* value (overall, log-rank)
**Colitis**	No irAE	27	2 (1-8)	
	Any other irAE	76	5 (3-11)	**0.03**
	Colitis	54	6 (5-12)	
**Musculoskeletal irAE**	No irAE	27	2 (1-8)	
	Any other irAE	101	5 (4-8)	**0.0054**
	Musculoskeletal irAE	29	12 (5-NA)	
**Pneumonitis**	No irAE	27	2 (1-8)	
	Any other irAE	115	7 (5-11)	**0.028**
	Pneumonitis	15	4 (3-NA)	
**Neurological irAE**	No irAE	27	2 (1-8)	
	Any other irAE	126	6 (5-10)	**0.029**
	Neurological irAE	4	3 (1-NA)	
**Myocarditis**	No irAE	27	2 (1-8)	
	Any other irAE	126	6 (5-10)	**0.0032**
	Myocarditis	4	1.5 (1-NA)	
**Cutaneous irAE**	No irAE	27	2 (1-8)	
	Any other irAE	69	5 (3-11)	**0.01**
	Cutaneous irAE	61	7 (5-24)	
**Endocrine irAE**	No irAE	27	2 (1-8)	
	Any other irAE	86	5 (4-8)	**0.0056**
	Endocrine irAE	44	8 (6-24)	
**Hepatitis**	No irAE	27	2 (1-8)	

PFS		N	Median PFS in months (95% CI, *p* value)	*p* value (overall, log-rank)
	Any other irAE	88	7 (4-12)	**0.027**
	Hepatitis	42	5.5 (3-8)	
**Other irAE**	No irAE	27	2 (1-8)	
	Any other irAE	89	6 (4-10)	**0.031**
	Other irAE	41	5 (4-17)	

Supplementary Table S7. Tabular overview of median OS outcomes as shown in Figure 2.

OS		N	Median OS in months (95% CI)	*p* value (overall, log-rank)
**Colitis**	No irAE	27	6 (4-30)	
	Any other irAE	76	NA (19-NA)	**<0.0001**
	Colitis	54	37 (23-NA)	
**Musculoskeletal irAE**	No irAE	27	6 (4-30)	
	Any other irAE	101	29 (23-NA)	**<0.0001**
	Musculoskeletal irAE	29	NA (NA-NA)	
**Pneumonitis**	No irAE	27	6 (4-30)	
	Any other irAE	115	37 (23-NA)	**<0.0001**
	Pneumonitis	15	46 (20-NA)	
**Neurological irAE**	No irAE	27	6 (4-30)	
	Any other irAE	126	46 (24-NA)	**<0.0001**
	Neurological irAE	4	20.5 (1-NA)	
**Myocarditis**	No irAE	27	6 (4-30)	
	Any other irAE	126	46 (26-NA)	**<0.0001**
	Myocarditis	4	1.5 (1-NA)	
**Cutaneous irAE**	No irAE	27	6 (4-30)	
	Any other irAE	69	32 (23-NA)	**<0.0001**
	Cutaneous irAE	61	50 (24-NA)	
**Endocrine irAE**	No irAE	27	6 (4-30)	
	Any other irAE	86	26 (19-NA)	**<0.0001**
	Endocrine irAE	44	NA (46-NA)	
**Hepatitis**	No irAE	27	6 (4-30)	
	Any other irAE	88	32 (23-NA)	**<0.0001**
	Hepatitis	42	50 (33-NA)	
**Other irAE**	No irAE	27	6 (4-30)	
	Any other irAE	89	32 (21-NA)	**<0.0001**
	Other irAE	41	NA (26-NA)	
